# Functional genomics of the digestive tract in broilers

**DOI:** 10.1186/s12864-018-5344-z

**Published:** 2018-12-13

**Authors:** Amélie Juanchich, Christelle Hennequet-Antier, Cédric Cabau, Elisabeth Le Bihan-Duval, Michel J. Duclos, Sandrine Mignon-Grasteau, Agnès Narcy

**Affiliations:** 1 BOA, INRA, Université de Tours, 37380 Nouzilly, France; 2 SIGENAE, GenPhySE, Université de Toulouse, INRA, INPT, ENV, Castanet Tolosan, France

**Keywords:** Chicken, Broiler, Digestive tract, Transcriptome, Gizzard, Intestine

## Abstract

**Background:**

The sustainability of poultry farming relies on the development of more efficient and autonomous production systems in terms of feed supply. This implies a better integration of adaptive traits in breeding programs, including digestive efficiency, in order to favor the use of a wider variety of feedstuffs. The aim of the project was to improve the understanding of genes involved in digestive functions by characterizing the transcriptome of different sections of the digestive tract: the junction between the proventriculus and the gizzard, the gizzard, the gastroduodenal junction, and the jejunum.

**Results:**

Total RNA from the four tissues were sequenced on a HiSeq2500 for six 23-day-old chickens from a second generation (F2) cross between two lines that were divergent for their digestive efficiency (D+/D-). Bioinformatics and biostatistics analyses of the RNA-seq data showed a total of 11,040 differentially expressed transcripts between the four tissues. In total, seven clusters of genes with markedly different expression profiles were identified. Functional analysis on gene groups was performed using “Gene Ontology” and semantic similarity. It showed a significant enrichment of body immune defenses in the jejunum, and an enrichment of transcriptional activity in the gizzard. Moreover, an interesting enrichment for neurohormonal control of muscle contraction was found for the two gizzard’s junctions.

**Conclusion:**

This analysis allows us to draw the first molecular portrait of the different sections of the digestive tract, which will serve as a basis for future studies on the genetic and physiological control of the response of the animal to feed variations.

**Electronic supplementary material:**

The online version of this article (10.1186/s12864-018-5344-z) contains supplementary material, which is available to authorized users.

## Background

Feed has represented the major proportion of production costs for meat-type chickens in recent years [1]. Additionally, the increasing demand for poultry meat and consequently for crops has accentuated the competition between animal and human consumption. Poultry breeding has until now favored highly performing animals that also need high quality resources and an optimized production environment to express their genetic potential. Currently, the evolution towards more sustainable livestock systems implies limiting inputs and to making use of the adaptive capacity of the animals to changing and even unfavorable dietary conditions. This requires a better understanding of adaptation processes - especially those related to digestive efficiency - in order to improve poultry breeding schemes. Using high quality feedstuff, which are easily digested by all birds, does not make it possible to distinguish birds with a high or a low capacity for digestion. Feeding birds with wheat-based diets instead of corn-based diets is a way to challenge their digestive efficiency in order to characterize their ability to digest various types of feedstuffs. A divergent selection experiment on the digestive efficiency of the chicken [2] using a wheat-based challenge diet led to marked differences in morphology and histology of the gizzard and small intestine [3]. The transit time between the different sections of the digestive tract may also explain differences in digestive efficiency: for instance particles, regardless of the size, spent 10 times less time in the gizzard of birds with low digestive capacity compared to birds with high digestive capacity [4]. The size and weight of the gizzard and the jejunum are highly different between birds as well [5, 6]. These results suggest that several functions are expected to be involved in the control of digestive efficiency.

The objective of the project was thus to identify genes associated with underlying mechanisms by characterizing the transcriptome of key specialized sections of the digestive tract: the gizzard (grinding and pre-digestion activity), the jejunum (major nutrient absorption site), and the junctions at the entrance and exit of the gizzard (regulation of motility, secretion and trophic activity of the digestive tract). The identification of genes and networks of genes involved in digestive processes is a prerequisite for understanding this complex biological function. These results will facilitate the taking into account of digestive genetics in selection schemes through the future identification of genetic markers or biomarkers of feed efficiency. This will also be useful for the evaluation of new breeding or feeding systems.

## Results

### Expression profiles of the digestive tract genes

#### Transcriptome analysis by RNA-seq

**Fig. 1:**
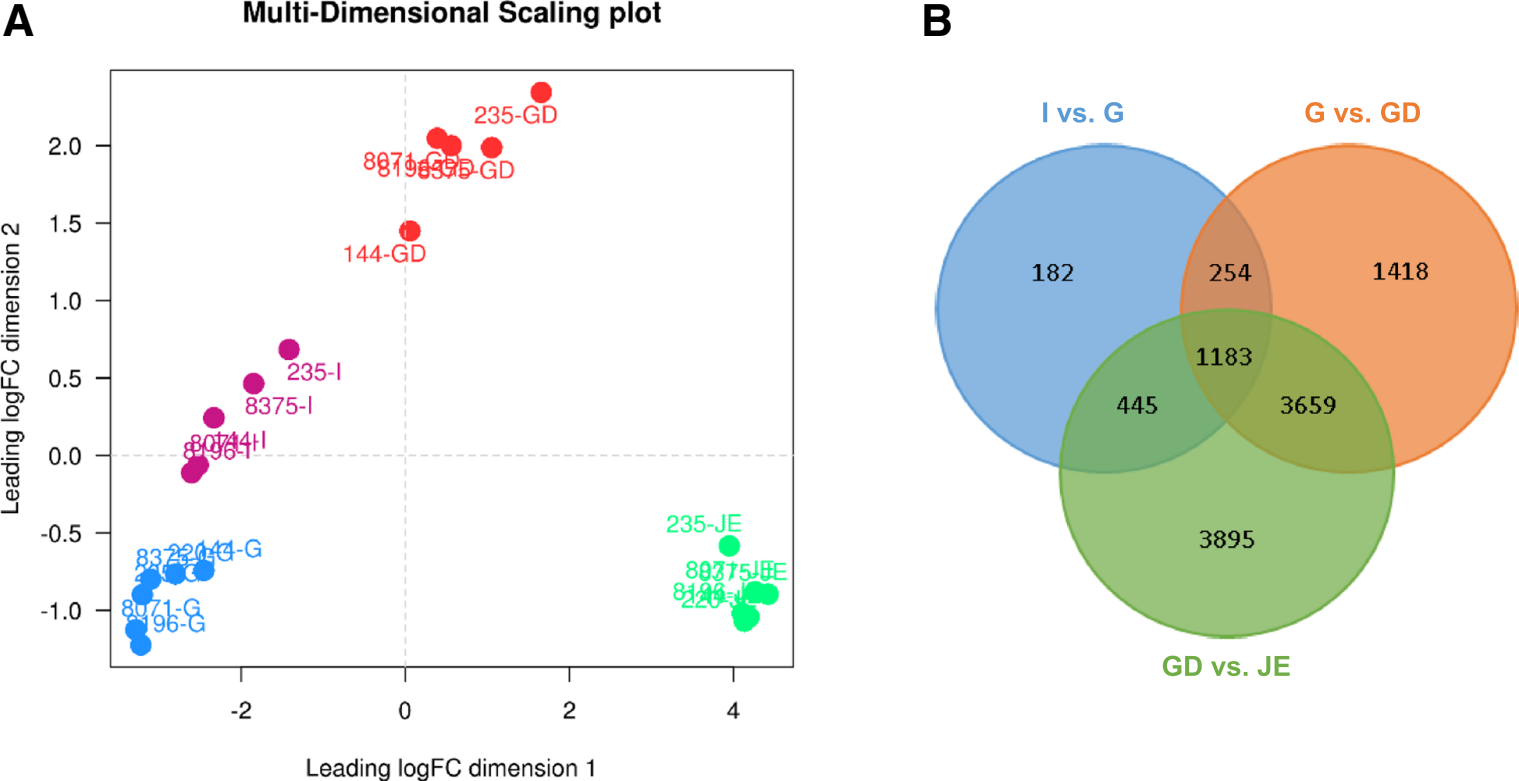
Transcriptome analysis: overlap between samples. **a** Multi-Dimensional Scaling (MDS) plot showing similarity between samples. Numbers refer to individuals (144, 220, 235, 8071, 8196, and 8375) and letters to tissues (I: isthmus, G: gizzard, GD: gastro-duodenal junction, and JE: jejunum). **b** Venn diagram showing the number of transcripts differentially expressed that are common between two or more comparisons (I vs G: isthmus vs gizzard, G vs GD: gizzard vs gastro-duodenal junction, and GD vs JE: gastro-duodenal junction vs jejunum)

**Fig. 2:**
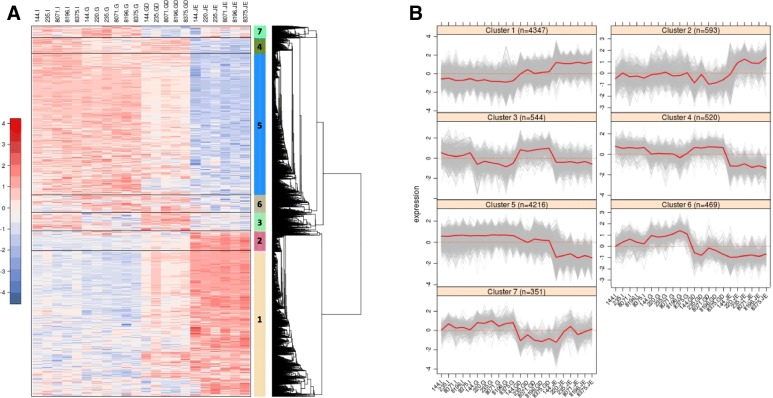
Expression profiles of differentially expressed genes between gizzard, its two junctions and jejunum. **a** Hierarchical clustering of the differentially expressed transcripts. Modular break of the hierarchical clustering defined 7 clusters of transcripts, numbered 1 to 7 according to (**b**). For each sample, numbers refer to individuals (144, 220, 235, 8071, 8196, and 8375) and letters to tissue (I: isthmus, G: gizzard, GD: gastro-duodenal junction, and JE: jejunum). For each transcript, the expression level (log2 count per million) is indicated using a color density scale (B) Expression profiles of transcripts within the 7 clusters. The red line indicates the average expression of the transcripts within the cluster. The size of each cluster is indicated in the title of each panel (n=). Numbers refer to individuals (144, 220, 235, 8071, 8196, and 8375) and letters to tissue (I: isthmus, G: gizzard, GD: gastro-duodenal junction, and JE: jejunum)

Sequencing of the 24 samples on Hiseq2500 generated between 5.2 and 10 million sequences per sample. TopHat2 [7] was used to align 89% of the reads to the Galgal4 version of the chicken genome. In total, 56,469 transcripts were reconstructed using the Cufflinks tool [8] and then quantified using featureCounts [9] on all 24 samples. A total of 15,396 transcripts were considered to be expressed in at least one of the four tissues. Biostatistical analyses with the edgeR package from the Bioconductor project [10, 11] revealed that no transcripts were found to be differentially expressed between animals with high or low digestive efficiency in any of the four tissues. Therefore, a focus on the differences between tissues was made and biostatistical analyses with the edgeR package revealed 11,040 differentially expressed (DE) transcripts between the four tissues, by pairwise comparisons. The transcriptomic analysis confirmed that the four tissues (I: isthmus, G: gizzard, GD: gastro-duodenal junction, and JE: jejunum) were clearly different compared to one another (Fig. 1a), and that the jejunum was the most different tissue compared to the three others. The homogeneity within each tissue was high, especially for the gizzard and the jejunum. The higher heterogeneity of the two junctions could partly result from the technical difficulty associated with their dissection. This descriptive analysis also prompted us to exclude two samples from one individual, which were clearly mislabeled (the isthmus and gastro-duodenal junction were possibly switched). Pairwise comparisons showed a significant number of genes differentially expressed between the four tissues. A total of 1273 transcripts were common between all pairwise comparisons. The Venn diagram in Fig. 1b focused on the spatio-temporal comparisons (I vs. G, G vs. GD, and GD vs. JE) and showed that 1183 transcripts (i.e. 11% of the total) were differentially expressed in the three comparisons. An increasing number of differentially expressed genes were found while moving forward in the digestive tract: 2064 transcripts were differentially expressed between the isthmus and the gizzard, 6514 between the gizzard and the gastro-duodenal junction and 9182 between the gastro-duodenal junction, and the jejunum. This could be linked to the evolution of the biological function of these tissues, with the jejunum having a different function than the three other tissues. Differentially expressed transcripts in at least one comparison were reordered to correspond to the hierarchical clustering results on their expression profiles displayed in a heatmap (Fig. 2a). The modular break in the hierarchical tree using the Dynamic Tree Cut package [12] identified 7 classes of transcripts, with very different expression profiles (Fig. 2b). Consistent with the previous observation (Fig. 1b), the greatest differences in expression were related to the higher (cluster 1 and 2) or lower (cluster 5) expression of genes in the jejunum, which represented 83% of the differentially expressed transcripts (9156/11,040). This strong impact of the jejunum in the results is consistent with the already known differences in functions between the four studied tissues. Indeed, the main function of the gizzard is mechanical grinding and pre-digestion of the feed, while the junctions upstream and downstream of the gizzard regulate the transit of feed into the gizzard. In contrast, the main function of the jejunum is the absorption of nutrients and immune system activity. Hierarchical clustering also revealed specific signatures of genes in the junctions upstream and downstream of the gizzard. Clusters 3 and 4 in particular contained 1064 genes specifically overexpressed in these two sections. The last two clusters grouped together transcripts that were overexpressed in the gizzard.

#### RNA-seq validation by RT-qPCR

In order to validate the RNA-seq experiment (RiboNucleic Acid – sequencing), 17 transcripts were chosen and their expression measured by RT-qPCR (Real-Time quantitative Polymerase Chain Reaction). Six invariant transcripts from the RNA-seq analysis were found to be stable across samples by RT-qPCR, so that the geometric mean of their expression was used as a reference transcript for the experiment [13]. The gene expression data obtained by two methods (RNA-seq and RT-qPCR) were highly correlated with Pearson correlations between 0.93 and 0.99 for the 11 genes that were measured (Table 1). The correlation coefficients were high for either weakly expressed genes such as *GDF10* (R^2^ = 0.978) or *MSMB* (R^2^ = 0.959) or for highly expressed genes such as *GKN1* (R^2^ = 0.996), thus validating the quantitative determination of gene expression levels by RNA-seq in the present study.

**Table 1:** Correlation between RNA-seq and RT-qPCR for 11 genes

Gene name	Pearson correlation
GRIK1	0.989
MYLK	0.996
GDF10	0.978
GKN1	0.996
NFKBIZ	0.987
LIPG	0.988
MSMB	0.959
SLC41A2	0.992
TM4SF4	0.994
CD151	0.966
MRPS26	0.934

Pearson correlations between RNA-seq and RT-qPCR analysis for the 11 studied genes. Correspondences between gene name used in this table and unique Ensembl ID are listed in Table 3

### Annotation and functional enrichment

**Fig. 3:**
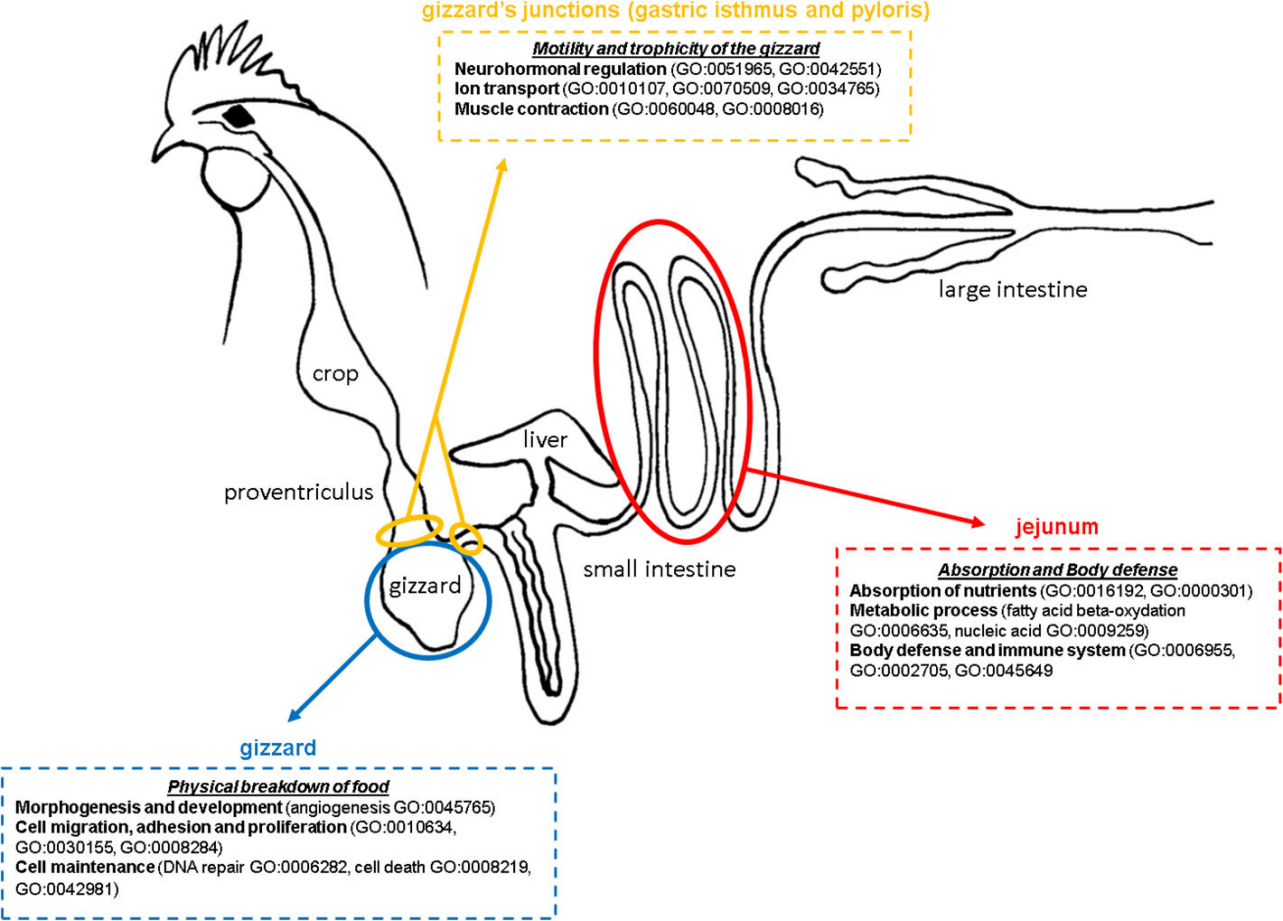
Representation of the molecular portrait of the digestive tract in broilers from results of this study

All expressed genes (differentially expressed or not) were annotated by Gene Ontology (GO) for Biological Process [14–16]. Of the 12,656 expressed genes, 6668 possessed at least one functional GO term in the Ensembl 88 database (53%). Enrichment tests were performed independently for each cluster of differentially expressed genes sharing similar expression profiles (Additional file 1). The expressed genes dataset was used as a background for the analysis. A total of 740 GO terms were enriched in at least one gene cluster and, among them, 695 were unique (Table 2). This shows that gene clusters are very different from one another, and quite exclusive in terms of function. The number of enriched terms for each cluster ranged from 35 to 276. GO terms were organized using the topology of the GO graph structure [14]. Functional analysis of the differentially expressed genes clusters showed diverse types of enrichment (Fig. 3), either basic cellular processes or specific processes, such as fatty-acid beta-oxidation in the jejunum. It clearly draws a picture (Fig. 3) of the important functions involved in the four tissues. Specific enrichments of the expression clusters are discussed below. Degrees of enrichments were diverse, ranging from *p*-value < 0.01 to p-value < 10^− 9^ (Additional file 1). Most were around 10^− 2^–10^− 4^, as could be expected.

**Table 2:** Functional enrichment analysis

Gene cluster	Number of transcripts	Number of genes	Number of enriched GO terms	main enriched functions
Cluster 1	4347	3684	190	metabolic processes (protein, fatty acid, glucose…), fatty acid oxidation, transport (lipids, glucose…), immune system (innate and response to bacterium, T cell differentiation, cytokine production, leukocyte-mediated immunity)
Cluster 2	593	509	50	macrophage differentiation, vesicle-mediated transport
Cluster 3	544	470	49	regulation of contraction, vasculogenesis, neuron development and synapse assembly, ion transport (K^+^ and Ca^2+^)
Cluster 4	520	454	82	morphogenesis (epithelium and nephron), ion transport (Cl^−^)
Cluster 5	4216	3477	276	development and morphogenesis, cellular organization, signal transduction
Cluster 6	469	371	35	DNA damage and repair, protein ubiquitination
Cluster 7	351	302	58	transcription and translation regulation, cell death andapoptotic processes, cytoskeleton organization

Enrichment analysis was performed using the Ensembl database and Gene Ontology for Biological Process. Cluster numbers refer to groups of genes of similar expression profiles from Fig. 2a

## Discussion

Identification of genes and networks of genes involved in digestive processes is a prerequisite for understanding the complexity this biological function. Functional analysis of the seven transcriptional clusters revealed the main functions that are important in key specialized sections of the digestive tract. Although functional annotation of the chicken genome remains poor in comparison to model species such as the mouse, functional analysis is still relevant using orthologous relationships with well-annotated species.

A significant enrichment of metabolic processes (labels: “fatty acid metabolic process, GO:0006631”, nucleic acid metabolic process, GO:0009259, “fatty acid beta-oxidation, GO:0006635”, Additional file 1) is found in the jejunum (cluster 1). Fatty acid beta-oxidation was highly enriched in the jejunum (*p*-value < 10^− 9^). This enrichment was confirmed by the presence of most enzymes of the fatty acid beta-oxidation cycle in the differentially expressed transcripts list (Additional file 1): acyl-coA oxidase (*ACOX1*, *ACOX2* and *ACOX3*), enoyl-coA hydratase (*ECHDC2*, *EHHADH*, *HADHA*), dehydrogenase (*BDH2* and *HSD17B4*) and acyl-coA transferase (*ACCA1* and *ACAT2*). The enrichment was present in both organelles in which the beta-oxidation occurs, i.e. the mitochondria and the peroxisomes (*LONP2*, *PEX5*) [17, 18]. Moreover, there was also an enrichment for lipid transport in the jejunum. This included the transport of sterol and cholesterol (*STARD4*, *SCP2*, or *NPC2*), apolipoproteins (*APOA1*, *APOA4*, *APOA5*, and *APOB*), and sphingolipids (*SPNS3*). This can be linked to the lipid absorption that occurs in the intestine [19]. Moreover, this enrichment in transmembrane lipids can also be linked to cell turnover, which is more rapid in poorly efficient birds [5]. Interestingly, we did not observe enrichment for protein or carbohydrate absorption in the jejunum.

The second-most enriched function in the jejunum is related to immune defenses (labels: “immune response, GO:0006955”, “leukocyte-mediated immunity, GO:0002705”, “cytokine production, GO:0001819”). Indeed, the intestine, in general, plays a major role in immune defenses, as it is in contact with the feed and with the microbiota, and carries out diverse immune mechanisms [20, 21]. A relationship between immune system function and digestive efficiency was previously suggested in a QTL detection study performed on the same F2 crosses [22]. Most of those QTLs are located on chicken chromosome 16, which carries the major histocompatibility complex.

In addition to these functions, cluster 2 exhibited a specific enrichment in the jejunum for vesicle-mediated transport (labels: “vesicle-mediated transport, GO:0016192” and “retrograde transport, vesicle recycling within Golgi, GO:0000301”) and immune response through macrophages (label: “regulation of macrophages differentiation, GO: 0045649”).

The expression profile in cluster 5 was the opposite of cluster 1 (overexpression in the gizzard and junctions compared to the jejunum). Enrichment analysis showed that functions related to morphogenesis and development (for instance angiogenesis “regulation of angiogenesis, GO:0045765”), cell migration, adhesion and proliferation (labels: “positive regulation of epithelial cell migration, GO:0010634”, “regulation of cell adhesion, GO:0030155”, “positive regulation of cell proliferation, GO:0008284”) are overrepresented in this cluster. We also observed an enrichment for signal transduction in this cluster (labels: “signal transduction, GO:0007165” and “intracellular signal transduction, GO:0035556”). This is consistent with the main function of the gizzard, i.e. grinding. The gizzard must pick up mechanical signals (such as particle size and gizzard filling). After transduction, the signal modifies internal cellular processes such as motility, namely contraction of the gizzard to continue grinding particles or start grinding when new feed particles arrive.

Genes belonging to cluster 3 (overexpression in the two junctions) showed an enrichment in neuronal control (labels: “positive regulation of synapse assembly, GO:0051965” and “neuron maturation, GO:0042551”). Transit, which is under neurohormonal control, is a key regulator of digestive efficiency [4]. An enrichment for muscle contraction (labels: “cardiac muscle contraction, GO:0060048”, “regulation of heart contraction, GO:0008016”) and for ion transport was also observed (labels: “potassium ion import, GO: 0010107” and “calcium ion import, GO:0070509”). Heart and smooth muscles share common genes involved in contraction. It is not surprising that such genes are expressed in the two junctions, which control the entry and the exit of the feed in the gizzard. Both junctions are known to regulate gastric functions such as motility, secretion, and trophicity. The isthmus contains interstitial cells of Cajal (ICC), necessary for regulating gastrointestinal tract motility [23, 24]. The gastroduodenal junction contains many endocrine cells that produce and release somatostatin, gastrin, or neurotensin [23], which regulate gastric acid and pepsin release and gastric mucosa trophicity.

Cluster 4 exhibited similar expression profiles to cluster 3, with more downregulation in the jejunum and less in the gizzard. Specific enrichments in this cluster related to ion transmembrane transport, but concerned different ions (labels: “regulation of ion transmembrane transport, GO:0034765” and “chloride transmembrane transport, GO:1902476”). Among the genes involved in ion transport in expression clusters 3 and 4 (Additional file 1), we observed potassium (*KCNJ11*, *KCNJ15*, *HCN2*), sodium (*SCN3B*), chloride (*SLC26A5*, *CLCN4*), and calcium (*CACNA1D*, *TRPC4*) transporters, as well as non-specific transporters (*TRPM2*). *CACNA1D* exhibited a 5-fold difference in expression between the junctions and the two other tissues. Most of those transporters are known to play a role in muscle contraction [25, 26] and in transmission of action potential in neurons. For instance, *SLC26A5* responds to changes in intracellular chloride level, modulates cell length, and acts as a molecular motor molecule [27]. *CACNA1D* is involved in muscle contraction [28], and *HCN2* is involved in spontaneous rhythmic activity such as contraction [29]. All of those ion transporters are essential for the activity of the gizzard, whose role is to grind the feed, and for opening and closing the junctions. Moreover, it seems that the balance between calcium and chloride in the junctions is the key factor for the contraction of these sections. These observations are consistent with the specific role of these junctions in controlling gastric motility [30, 31].

Lastly, enrichment of genes involved in cell maintenance functions was observed in the gizzard, compared to the other studied sections (clusters 6 and 7). For example, DNA repair and damage were enriched in cluster 6 (labels: “regulation of DNA repair, GO:0006282” and “regulation of DNA damage checkpoint, GO:2000001”) and cell death and nucleic acid metabolism in cluster 7 (labels: “cell death, GO:0008219”, “regulation of apoptotic process, GO:0042981”, “regulation of transcription, GO:0006355”, positive regulation of transcription elongation, GO:0032968). This enrichment may be related to the high level of expression of GKN1 in the gizzard. Although there is considerable inter-individual variability, GKN1 represented 20 to 25% of the total gene count in the gizzard in the current study and was highly over-expressed in the gizzard compared to the jejunum (1000 times more). Gastrokine 1 seems to have a mitogenic activity and may play a role in the maintenance of the integrity of the gastric mucosal epithelium [32, 33].

## Conclusion

This analysis allows us to draw a first molecular portrait of the various sections of the digestive tract of chickens selected for their digestive efficiency. Genes differentially expressed in the four sections correspond to biological functions related to the motility and trophicity in the junctions upstream and downstream of the gizzard, physical breakdown of the feed in the gizzard, and absorption and body defense in the jejunum. This initial description will serve as a resource for future studies on the genetic control of digestive efficiency or its dietary regulation. It opens up prospects for identifying key genes involved in the control of digestive functions in chickens and ultimately for the emergence of new breeding or feeding strategies.

## Methods

### Animals and sample collection

Chickens from the D+ and D− lines that had been divergently selected for high or low digestive efficiency, respectively [2], at 3 weeks of age, were crossed at generation 8 of selection to produce an F2 design. The initial population, on which the selection experiment was performed, is a pure line of broilers used in a commercial crosses dedicated to medium-growing broiler production, reaching the 2 kg market weight at 7 weeks of age. Chickens used in the experiment were hatched and reared at INRA UE1295 PEAT (France). From hatch to 10 days of age, all birds were reared in one group on the floor, and then transferred to individual cages from 11 to 23 days of age. Throughout the experiment, the birds were fed a diet similar to that used during the selection experiment [2]. This diet included 55% Rialto wheat, which is very hard and viscous, and thus especially difficult to digest.

A total of 864 F2 birds were hatched for a QTL (Quantitative Trait Loci) detection experiment [22]. All F2 birds were recorded for feed efficiency at 2 weeks of age and AMEn (Apparent Metabolisable Energy corrected for zero nitrogen) was calculated for each bird at 3.5 weeks. For transcriptome analysis in the current experiment, six extreme birds were selected based on AMEn values. The tissues of interest were collected at the end of the balance trial in three individuals selected based on their high digestive efficiency (3 AMEn+, μ = 3521 kcal/kg of dry matter) and three for their low digestive efficiency (AMEn-, μ = 2610 kcal/kg of dry matter). Tissues from the digestive tract were sampled and immediately frozen in liquid nitrogen and stored at − 80 °C. The tissues included the gizzard (G), the jejunum (JE), and the two gizzard junctions: the isthmus (I, between the proventriculus and the gizzard), and the gastro-duodenal junction (GD, between the gizzard and the duodenum).

### Transcriptome analysis

Total RNA from the four tissues of six individuals was extracted with the RNeasy Mini kit (QIAGEN). The 24 RNA samples (6 animals × 4 tissues) were sequenced for 2x125pb on HiSeq2500 (GeT-PlaGe facility, Toulouse, France) and multiplexed according to the standard Illumina sequencing protocol.

The readings were aligned to the 4th version of the chicken genome (Galgal4) through TopHat2 (v2.0.14) [7] with default parameters (-N 2, −-bowtie2, −-library-type fr-unstranded). Cufflinks (v2.2.1) [8] was used for the assembly of the mapped reads and the resulting transcripts were quantified with featureCounts v(1.4.5-p1) [9], again with default parameters (annot.inbuilt = “gga4”, GTF.attrType = “gene_id”). Low counts were removed from the dataset (counts < 5 in at least a quarter of the samples, i.e. 6/24). Varying sequencing depths were taken into account in the model, with size factors calculated using the trimmed mean of M-values (TMM). Remaining transcripts define our set of expressed transcripts in the study. Biostatistical analyses of the RNA-seq data are based on a generalized linear model using the Bioconductor edgeR package (version 3.16.5) [10, 11]. The experiment was described in a complex design with two factors, digestive efficiency with two levels and tissue with four levels. No transcripts were found to be differentially expressed between animals with high or low digestive efficiency. Nevertheless, digestive efficiency structured our groups and therefore was kept in the model. To facilitate the pairwise comparisons between tissues, a group factor combining both digestive efficiency and tissue was only included in the model. Differentially expressed transcripts between tissues were identified by pairwise contrasts on the average digestive efficiency effect. *P*-values were adjusted by controlling the false positive rate below 0.05 with a Benjamini-Hochberg correction [34]. A hierarchical classification on expression data transformed with log2 count per million for differentially expressed transcripts between tissues was built based on a Pearson correlation and an average link aggregation distance. A modular break in the hierarchical tree using the Dynamic Tree Cut package [12] (version 1.63–1 with the following parameters: deepSplit = 3, minClusterSize = 300) was applied to find clusters of transcripts with similar expression profiles.

### RT-qPCR validation

Total RNA samples (2 μg) used for RNA-seq analysis were subjected to reverse-transcription, using Superscript III reverse transcriptase (Invitrogen, Cergy Pontoise, France) and random primers. A 1:25 dilution of the RT product was used for real time PCR amplification using a LightCycler 480 SYBR Green I Master (Roche Applied Science, Mannheim, Germany), as recommended by the manufacturer’s instructions. PCR conditions using the LightCycler 480 (Roche Applied Science, Mannheim, Germany) were as follows: a thermal denaturation step of the polymerase (95 °C/10 min) followed by 40 cycles of amplification (denaturation: 95 °C/10 s, annealing: 60 °C/20 s, and elongation: 60 °C/10 s) with measurement of the emitted fluorescence at the end of each cycle. A melting curve (60 °C to 95 °C) was also performed to verify the presence of a single product with a specific melting temperature. Each run of PCR consisted of triplicate samples, and contained “no template” controls without cDNA (complementary DesoxyRiboNucleic Acid). A standard curve was determined using serial dilutions of a pool of 24 RT products. Calculation of mRNA (messenger RNA) levels was based on the detection of the threshold cycle and the PCR efficiency derived from the standard curve. To account for variations in RNA extraction and reverse transcription reactions, RNA levels were corrected with the use of reference transcripts [13]. In order to validate the RNA-seq experiment, 17 transcripts were chosen for determination by RT-qPCR, based on their expression profiles. Among the 17 transcripts, 6 were chosen as invariant among the samples and set as reference transcripts (*SMARCB1*, *MATR3*, *HNRNPA3*, *MAU2*, *STAG2* and *EIF3l*). The 11 others were chosen from the 7 different clusters of transcript expression, with various levels of expression (*GRIK1*, *MYLK*, *GDF10*, *GKN1*, *NFKBIZ*, *LIPG*, *MSMB*, *SLC41A2*, *TM4SF4*, *CD151*, and *MRPS26*). All primers used in the study are listed in Table 3.

**Table 3:** Primers used in the experiment

Gene name	Gene ID	Transcript ID	Forward primer	Reverse primer
SMARCB1	ENSGALG00000005983	ENSGALT00000009622	AGAAGCCCGTCAAGTTCCAG	TGTGGCTAGTCGTCTCCAGA
MATR3	ENSGALG00000002478	ENSGALT00000003907	ATTCACAAGGTCATGGGCGT	CCTTCCAAGAGATGCTGGCA
HNRNPA3	ENSGALG00000009250	ENSGALT00000038715	ACTCTTGCGTGGAAGAGGTG	TTTACTGTGAGATGCGCCCC
MAU2	ENSGALG00000002969	ENSGALT00000004696	GAGTGTGAAGCCGTGTCTGA	GGGCAGCCAGTGAAAGAGAT
STAG2	ENSGALG00000008482	ENSGALT00000013823	GCACACACCAGTCATGATGC	TGGTGTTCAGGCTGCATAGG
EIF3I	ENSGALG00000003337	ENSGALT00000005281	GACATGTGCTCACTGGCTCT	CACTGCTGAGCTGGTCTTCA
GRIK1	ENSGALG00000015835	ENSGALT00000025530	CCCTTCATGACGCTGGGAAT	GGACACAACTGACTCCGAGG
MYLK	ENSGALG00000011708	ENSGALT00000019136	TGCTGCTAGGTTTGACTGCA	GGAAGTGACGGGACTCCTTG
GDF10	ENSGALG00000005985	ENSGALT00000009625	TGCTGAGCTTGATTCTGGGG	GCCACACTGTTAGGTTCGGA
GKN1	ENSGALG00000000114	ENSGALT00000000163	ATCACCATCAACGTTGGCCT	GTTGTCCATGCGTTCTCAGC
NFKBIZ	ENSGALG00000015346	ENSGALT00000024766	CCAGCCCTGTTTCCCTGAAT	CGGACTGTCGTGGTATTGCT
LIPG	ENSGALG00000002712	ENSGALT00000004279	CCTGCTGGCCCTATGTTTGA	GATCCCAATGCTGACACCCA
MSMB	ENSGALG00000020840	ENSGALT00000033414	GACTGCTTAGAGTGCTCCTGT	TTGAGTGGTCGGCTTTCTCC
SLC41A2	ENSGALG00000029019	ENSGALT00000045165	GGCCACACTTCCTTAACTCCA	TGCACCATCCAGTCAGCAAT
TM4SF4	ENSGALG00000010427	ENSGALT00000016979	TATTGGGATCTGGCGTGCTG	CGCAAACCTCTTTCCACAGC
CD151	ENSGALG00000006856	ENSGALT00000039051	CAGGGGTGGTTGTGATGGTT	GGCCAGGATTCCAGCAATGA
MRPS26	ENSGALG00000014118	ENSGALT00000037114	TCCATCTTCAGGTCCGAGGT	CTCAGCATCATTCCAGGCCA

### Functional analysis

The correspondence between expressed transcripts and gene annotation was done using the Ensembl Genes 88 database [35, 36] to perform functional analysis at the gene level. Then, differentially expressed genes between the 4 tissues were annotated by Gene Ontology [14, 16] for Biological Process as GO terms using the R package TopGO from the Bioconductor project [37] and the Ensembl Genes 88 database as a reference [35, 36, 38, 39]. Enrichment for specific functions within each cluster of genes with similar expression profiles (based on associated transcripts expression level) was tested using a Fisher’s exact test and “elim” algorithm (*p* < 0.01) implemented in TopGO. A dataset of all expressed genes was used as a background for functional enrichment tests.

## Additional file

Additional file 1: Functional enrichment analysis: 635 Biological Process enriched GO terms. (XLS 2148 kb)

## Abbreviations

AMEn: Apparent Metabolisable Energy corrected for zero nitrogen
cDNA: Complementary DesoxyRiboNucleic Acid
GO: Gene Ontology
MDS: Multi-Dimensional Scaling
mRNA: Messenger RNA
QTL: Quantitative Trait Loci
RNA: RiboNucleic Acid
RT-qPCR: Real-Time quantitative Polymerase Chain Reaction
